# Abstracts and Gleanings

**Published:** 1888-04

**Authors:** 


### ABSTRACTS AND GLEANINGS.

**The Treatment of Intermittent Fever.**—Robert C. Kenner, A. M., M. D., in *American Practitioner and News*, says: The treatment of intermittent fever calls for remedies directed to the relief of the issues of the paroxysm it elf, and the prevention of future attacks.

Sternberg says: "The sensations of the patient during the *cold stage* seem to turnish an indication for the *treatment*." He advises against the piling on of excessive bed-clothing, and says truly, patients should be lightly covered, and only sufficiently to protect from draughts. Hot bottles should be applied to the feet and warm irons laid against the spine. Very little water or other fluids should be allowed in this stage. Even warm drinks, recommended by some authors, have often proved harmful in my hands by exciting and vomiting soon after being swallowed, or later on in the paroxysm. Besides these measures I administer a full dose of opium by the mouth or hypodermically. When we find our patient in the cold stage of the fever, which is more protracted or severe than the former paroxysms, dependence should be placed in chloroform in preference to opium. I rarely use it, however, when the paroxysm is light, but reserve it for those cases which border on the pernicious forms of this fever. I usually administer it in doses of one dram in emulsion. Even when the cold stage has advanced considerably, the abortive energies of chloroform generally display themselves. If a single dose fail, I have no hesitancy in repeating it in forty minutes. The second dose usually succeeds in producing sleep.

Treatment of the *hot stage* is very often imperative. It is frequently complicated with excessive gastric irritability, more or less cerebral congestion, delirium, etc. The febrile action often runs to a dangerous point, and this will demand treatment. For the protracted vomiting which often occurs, oxalate of cerium was in a great many cases successful; I order in five-grain doses repeated hourly. When this alone fails I order cracked ice as an adjuvant. Yet there often occur cases in practice which will not yield readily to any treatment. These are sometimes cured by hydrarg. chlo. mit., in doses of one-half grain, repeated hourly or every half hour. When this fails I use dry cups, applied over the epigastrium, and give hypodermic injections of morphia, and generally this troublesome symptom subsides. For the headache and threatened or incipient cerebral hyperemia, I order bromide of potassium in doses of from thirty grains to one dram, repeated if necessary in four hours. It is advantageous as soon as there is any headache, to apply cloths wet with cold water to the head. When the temperature is very high, abve 107° F., the cold bath is indicated, and should be used.

*The means of preventing future paroxyms.*—Iodine in some cases is probably of value. Sternberg and other good observers have found its field of usefulness quite small. My own use of this agent has been attended with results that are against the antiperiodic virtues it is alleged to possess. I do not consider it a remedy that can be trusted.

The use of nitric acid, given three times daily in doses of ten drops diluted properly has, in the hands of Prof. W. A. Hammond, been followed with results that would recommend a more extended trial of this remedy.

Arsenic has long had a reputation in the treatment of this fever. It is often claimed that it is the most reliable agent when quinine fails.

It cannot be reasonably doubted that a decoction of lemons will cure a certain percentage of cases of intermittent fever. It is recommended by Maglieri, that an entire fresh lemon be cut in slices and put into an earthen vessel with eighteen ounces of distilled water and boiled for two or three hours, till sixteen ounces remain. After standing all night this is strained and given to the patient, who is to swallow it at several gulps. It is insisted that this be continued for ten or fifteen days.

Piperine exerts a curative influence in some cases. There is reason to believé it is of use in that form which is thought to be a *habit*. It produces a stimulating influence which is agreeable, and may be used in some cases of this type with confidence

I have used *Cornus florida* with an encouraging degree of success. It long ago had a reputation in the treatment of intermittent fever, but was, as many other remedies have been, almost abandoned. In four cases where quinine, on account of idiosyncrasy, was inadmissable, I prescribed it in doses of one dram of the fluid extract, to be given for five hours before the expected paroxysm, or one dram every hour until four doses had been taken.

According to several observers eucalyptus is a remedy of merit. Hertz regards it as a "cheap substitute for quinine." It seems to me to be more a tonic than any thing else, and it has been used in the mild or *habit* type of intermittent fever more than anywhere else.

Phosphorus has been used by a provincial Russian practitioner with an encouraging degree of success.

James Currie first introduced water into the treatment of this and other fevers. Its field of usefulness is now pretty well understood. It is useful when the fever is the *habit* type. I order the patients to take a cold shower bath an hour before the expected paroxysm. These baths should be followed with rubbing, and ought to be taken daily for two weeks.

The picrate of ammonia has recently been introduced by Dr. Martyn Clark, of India, who reported the results of his use of the drug to the London *Lancet*. In the last four years Dr. Clark has treated ten thousand cases with the "happiest results." Relative to the method of administration Dr. Clark says : "I usually give it in doses of from one-eighth of a grain to a grain and a half four or five times a day, in pill. Half a grain is a fair average dose. This given, the result is soon visible.

There is little reason to expect much from carbolic acid. Reports placing its antiperiodic properties in a bright light are not to be taken to mean always what they say.

Salicylic acid has also proved worthless in this disease in my hands. Coffee is another article one comes across in the literature of intermittent fever. Prefessor Paili, of Milan, found the sulphite of magnesia to be of value in the treatment of this disease. The late Professor Flint found it, after a thorough trial, wanting, and other observers have had like experience

In colocynth we have a remedy suitable in cases attended with marked biliousness.

The remedy which justly claims at the hands of the profession more esteem, and having the widest range of usefulness, beyond question is quinine and the other alkaloids of the cinchona barks.

Salicin I have never used to any extent, and the evidence of its value in this disease is of a doubtful nature. So, also, are we to regard gelsemium and many other drugs. They possess possibly some feeble antiperiodic powers or exert their curative energies by action on the nervous system.

**Bi-Borate of Ammonium in Uric Acid Calculi.**—Dr. J. R. Crittenden, Unionvil e, Va., in *Virginia Medical Monthly*, says: The treatment of paroxysm of "renal colic" is sufficiently plain, and need not be here recapitulated. But the preventive of these paroxysms is a matter which has puzzled the master minds of the medical profession from time immemorial.

It is almost useless for me to say that without a chemical examination of the urine—the sediment, and particularly the little calculi—the treatment will be a mere matter of guess-work. There are cases of renal colic dependent upon uric acid calculi, recurring several times a day, once in two or three days, once a week, once a month, etc. I find the bi-borate of ammonium to be a drug possessing a peculiar influence over the calculi in question.

In patients who have these attacks of renal colic every day, I prescribe 20-grain doses of ammonium bi-borate every two hours until a free passage of urine takes place; then every four hours until all feeling passes away; then I decrease to 15-grain doses three times a day before meals in a glass of flaxseed tea, and so continue for several months—discontinuing it for a day or two at a time every two weeks. In cases in which the paroxysms recur once a week, I pursue the same plan of treatment.

Now, if these paroxysms recur once a month, I give 15 grains before each meal for two or three months at a time. I find it a good plan, when we give it for a length of time, to combine with it lithiated extract hydrangea in teaspoonful doses. I will append two cases:

1. Mr. A., age 52; suffered with attacks of renal colic once or twice a month. I gave him:

℞. Bi-borate of ammonium........................℥ j,
Water........................................O j.

M. S.—Take one teaspoonful before each meal in flaxseed tea.

I continued this line of treatment for two months with perfect relief.

2. Mrs. W., age 43; suffered intensely with renal colic. I procured a sample of her urine and tested it, and found it contained a number of small uric acid calculi. I gave her of a solution like the above, 1 tablespoonful every two hours until she was entirely relieved. I then gave her 15 grains every four hours for a week, followed by the following:

℞. Bi-borate of ammonium ............................ ʒ v,

Lithiated ext. hydrangea ............................ ℥ iv.

M. S.—Take one teaspoonful just before each meal in flaxseed tea.

I continued this line of treatment for two months with gratifying results and perfect relief.

I always find the solution to be best when made fresh.

**Treatment of Sciatica.**—Dr. Metcalf, of New York, says that no prescription for sciatica has ever equalled in efficacy the following:

℞. Tinct. aconit. rad ..................................

Tinct. colchic. sem ..................................

Tinct. belladonna .................................. aa ʒ j.

M. Sig.—Dose, 6 drops every six hours.

He also uses triturate tablets, each containing 3 drops of the following:

℞. Tincture of aconite root ......................
Tincture of seeds of colchicum ..............
Tincture of belladonna ......................
Tincture of actea racemosa . . aa equal parts by volume.

Dose, 1 every four or eight hours.—*Eastern Medi. Journal.*

**Coca.**—Coca, the chief alkaloid of which (cocaine) has recently proven to be of so much value as a local anæsthetic, should not be confounded with coca, the plant from which chocolate is derived, and which is an entirely different plant.

Coca is a shrub, native of South America, especially Peru, Bolivia, and adjoining states, where it grows wild, and is also largely cultivated on extensive plantations. It is propagated from the seed, and grows to a height of from four to six feet, has a leaf similar to the tea leaf, begins to yield within two years, and continues productive many years. The leaves are the part used; they are stripped from the branches several times a year, and after careful drying in the sun, are packed in bundles for future use.—*Journal of Materia Medica.*

**Guacal.**—Guacial is an active agent of creasote. Sabi believes it to be more efficient than creasote, and less likely to be impure. The dose is ¼ to ½ a drop.

**Therapeutics of Neuralgia of the Head.**—Eulenburg emphasizes the great value of massage in the various neuralgic affections of the head. The affected parts are both to be beaten rapidly (tapotement) and to be gently stroked (effleurage). Besides, even if the seat of the affection be in the forehead, the occipital region is to be likewise treated. Etilogical factors, such as obesity or heart disease, are to receive due attention. Internally antipyrin, in 15 grain doses, is the most efficient remedy. Galvanization is often useful.—*Ex.*

**The Value of Acetanilid in Enteric Fever.**—During the present autumn I have treated in private practice thirteen cases of enteric fever after the following general plan of treatment, with some slight variations to suit individual cases. When seen during the first few days of the disease, a sharp mercurial parge was induced, and afterward, when necessary, the frequency of the motions was regulated by the administration of opium. This, however, was rarely the case, as in the majority of the cases of enteric fever met with here the diarrhœa does not often become so aggravated as to demand treatment specially directed to it. If not seen early, the mercurial was omitted. From the establishing of the diagnosis until the subsidence of the fever, quinine was given in two grain doses every four hours. Antifebrin was given as follows: in eight cases, two grain doses were administered at 1 p m. and again at 5 p. m In two of these cases it was found necessary to give another two grain dose at 9 p. m. In three cases the antifebrin was given in one and a half grain doses at 1 p. m. and at 5 p. m ; and in one of them the same amount was given at 9 p. m., One of the two remaining cases had three grain doses at 1 p. m., and at 5 p. m.; the other received two one grain doses per day at 1 p. m., and at 5 p. m. In about one-half of the cases cold sponging was resorted to from three to six times a day.

The highest point of febrile range reached in the evening exacerbation of any of the cases was 106.2° F.; the lowest 103·5° F.; the average temperature in the evening exacerbation of the cases was 104.4° F. This observation was made in each case on a day at the close of the second week, when the disease was at its height; the antifebrin being suspended one day for this special purpose.

All of the cases made good recoveries; the average duration of the fever was twenty-two days. The patients were all young persons, ranging in age from fifteen to thirty-seven years; eight were males and five were females. In none of the cases were the slightest evil effects observed to follow the use of the acetanilid, save in a single instance where the patient complained of a slight degree of chilliness coming on soon after the drug was taken. In this case after reducing the dose from two grains to half that amount the temperature was satisfactorily reduced and the chilliness was not experienced. In every case the use of the drug in the doses indicated above invariably wrought a reduction in the temperature of from 1.8° F. to 3.4° F.

*Per contra*, I found that, regarding the cardiac condition, less need for the use of alcoholics arose during the progress of the disease than in cases treated after the most approved methods of a year or two past. In addition to this the degree of muscular prostration and exhaustion was not so marked as I have noticed when pursuing the methods of treatment recommended in our standard text books. These results are, of course, readily explained by the efficient action of the acetanilid in lowering the temperature, thereby lessening the tendency to, and the amount of. granular degeneration of the muscular tissues very much more than in cases treated by the older methods, under which the febrile range could not be so readily controlled.

In conclusion the following advantages may, I think, be claimed with justice for antifebrin over all other antipyretics in the management of the hyperpyrexia of enteric fever.

1. The size of the dose is small, and from the bland and unirritating character of the drug is easy of administration and not liable to produce gastric irritation.

2. The happy effects of the drug in reducing hypernormal temperature and in rendering the patient more comfortable by its soothing effects on the erethitic state of the nervous system accompanying febrile processes.

3. The absolute safety of acetanilid when used in medicinal doses. This conclusion may at present be called into question, but I am fully convinced that, when we cease to give our patients *toxic* doses of the drug cases of collapse and cardiac failure will cease to be observed.—*Dr. Way in Medical News.*

**Pleuritis.**—In a discussion on pleuritis in Marion County Medical Society, Dr. Ferguson said (*Indiana Medical Journal*):

In the treatment of acute pleuritis, thoracentesis is not done as frequently as it should be, and the result is that many cases are badly cured and subsequently die of empyema, or remain permanent invalids.

In thoracentesis four important points are to be considered:

1. When should we perform the operation?
2. At what point in the thoracic wall should we make the puncture?
3. How should the operation be performed?
4. How much fluid should be withdrawn?

In general the sooner the operation is performed after effusion has taken place the better. It is not necessary to wait till there is a large quantity of effused fluid, nor until there is great embarrassment in the respiration. The operation can easily be performed with a trocar and canula, with a rubber tube attachment, one end of which should be immersed in a basin of carbolized water. In this way the fluid can be withdrawn without the admission of air into the pleural cavity. Many authors recommend that the puncture should be made between the fifth and sixth ribs on the left side, and between the fourth and fifth on the right side; but I puncture posteriorly at the most dependent portion of the effused fluid, viz., between the ninth and tenth ribs It is not necessary to withdraw all the fluid, as it is well known that when a small quantity only is withdrawn, absorption is at once set up and goes on rapidly. The majority of cases of acute pleuritis, if seen at the commencement, can be aborted with a fly blister, opium and aconite.

Dr. J. H. Woodburn: We do not have as many cases of pleuritis now as in former years, for the reason that we have better houses, better food, and warmer clothing. In the early days we had what the authors described as bilious pleurisy, for which we used calomel freely. In acute pleuritis we used the lancet freely, followed by opium, and seldom failed to abort the disease. I would use blisters in the first stage, and believe that a little judicious blood-letting would still be a good remedy, although it is now out of fashion.

Dr. T. B. Harvey: If I see a case of acute pleurisy early in the first stage I can usually abort it with a hypodermic of morphine. As an arterial sedative I prefer aconite. Turpentine stupes are valuable local applications Aspiration is not necessary in all cases. When aspirating, the patient should be placed in the sitting posture, and the puncture made at the most dependent portion of the fluid. I would not blister in the early stages of the disease. Later, blisters promote absorption.

Dr. G. J. Cook: Many cases can be aborted by counter irritation at the beginning of the attack. That pleuritis is a very common disease can be demonstrated in any dissecting-room. In my experience as demonstrator of anatomy, the majority of subjects were found to have old pleuritic adhesions.

Dr. J. H. Oliver: Had no faith in blisters. If he used them at all, he would apply them to the bottom of the feet to keep the patient in bed.

Dr. Blitz: Had seen cases aborted by staying the chest with adhesive plaster and the administration of morphia.

**How to Preserve Ligatures.**—Prof. S. W. Gross condemns the use of carbolized oil for preserving *catgut ligatures*, as it forms a nidus for germs. Ten per cent. carbolized water doses the same thing unless changed every two weeks. He recommends the following way to preserve them: Take the animal ligature, prepare a 1 to 5 aqueous solution of chromic acid:

Acid. chromic.............................℥ j,
Aquæ.....................................f℥ v.

M.

Add one ounce of the above solution to five ounces of glycerine, place the ligatures in this solution, and leave them there for one week ; then take them out of this solution and hang them up until they are perfectly dry. Placed in the following solution they will keep until needed :

Alcohol.............................parts 15
Glycerin............................parts 1
Acid. carbolic......................10 %

M.

If thrown into a 1-1000 solution of corrosive sublimate a few minutes before using, they will become soft and pliable.—*College and Clinical Record*, February 1888.

**Ergot in Obstetric Cases.**—A writer in *Medical Brief* says: I have been a colaborer in the healing art for thirty-one years, and, with a liberal share of obstetrical practice, think I have learned a few facts. In all my practice, I have only found it necessary to resort to the forceps three times, and my process of action is, as a rule, that when I find the os dilated to the size of a half dollar, and the os soft and yielding, with irregular pains, showing a disposition to linger or become tedious, I never hesitate to administer ergot with the most satisfactory results. My plan is as follows: I have about three ounces of black-pepper or ginger tea prepared, and to this tea I add a large teaspoonful of the freshly prepared fl id extract of ergot, then the patient is required to take this mixture in three equal portions, a half hour between each dose. And in a large per cent. of the cases, labor will be completed in two hours from the administration of the first dose of tea and ergot, and a tonic condition of the womb has followed in three fourths of the cases that have come under my observation, as speedily as could be expected after the organ had undergone so terrible a change as it does in so short a time; one hour distended almost to its greatest capacity, and the next hour reduced to almost a normal size when unimpregnated. I have as yet to see the first evil result from the administration of ergot, when given as above directed.

The question might then be asked why give it in connection with the pepper or ginger tea? My answer is: I have found by long years of experience, that both pepper and ginger are prompt agents in arresting what are known as false or spurious pains. I employ ergot in small doses, where miscarriage is threatened, with satisfactory results. Therefore, I do not know how ergot acts in other doctors' hands, nor do I know what their plan of administering the drug is. I only know how I use it, and also what it has done when thus used. I would be very loth to give it up in obstetric practice.

**Toxic Effects of Iodoform Cutaneous and Systemic.**—Dr. R. W. Taylor, Surgeon to the Charity Hospital, New York, read a paper on this subject before the American Dermatolagical Association, at its eleventh annual meeting. The following is a su[illegible]-mary of his conclusions:

*A. Its use as indicated:* 1. On fresh wounds. 2. On diseased surfaces—gangrenous, chancroidal, phagedenic, syphilitic, tuberculous—and on those slow to take on healthy granulation. 3. On the surface of necrosed bone.

*B. Its use is contra-indicated:* 1. On freshly cut bone. 2. On granulating surfaces. 3. In cases in which it is known or is found to produce toxic effects.

*C. Modes of use:* 1. It should be dusted on the surface lightly and sparingly. 2. In wounded cavities or in the natural cavities as small a quantity as possible should be employed ; in the former it is preferable to use it in the form of gauze. 3 It should never be rubbed in with the finger. 4. Its application should be renewed as frequently as possible. 5. Such aids to absorption as tightly fitting bandages and impermeable dressings shou.d not be used. 6. *Its use shonld be discontinued as soon as healthy granulations appear*.. 7 It should not be used coincidently with other antiseptics. carbolic acid especially (Mcsetig-Moorhot). 8. It sho ıld be used with great caution in the young and the old ; in anemic and neurotic persons, and those suffering from weak heart or Bright's disease. Should toxic symptoms appear, the dressing must be promptly and thoroughly removed.—*New York Medical Journal.*

**Ergct and Acetic Acid in Post-partum Hemorrhrge.**— John A. Francis reports the following in the *British Medical Journal*, February 11th : It must have occurred to every one with a few years' experience in practica midwifery to have encountered cases of *inertia uteri* after delivery. The labor may have been normally rapid and strong, and the placenta and membra es discharged entire after the usual interval, leaving a firmly contracted uterus ; but presently, notwithstanding the continued pressure of the hand, the uterus elongates and acquires a featherbed feel, and, refusing again to contract, hemorrhage results ; or, the uterus may have been inert from the first, barely effecting delivery, or requiring instrumental interference ; and all this notwithstanding the previous administration of large doses ot ergot. In cases where the uterus contracts firmly at first, I take it to be a continuation of the routine of labor, and the uterus, finding no opposition and nothing to expel, relaxes instead of passing into a state of tonic contraction. I should like to insist on the value of a pasty or of a hectic complexion as a warning, and that an accelerated pulse, be it strong or weak, is an almost certain forerunner of hemorrhage.

The liquid extract of ergot is very unreliable in these cases, and I have been grievously disappointed with preparations of ergot and ammonia in several instances. I have been equally pleased at the quick action of vinegar given after ergot has failed, especially when followed by brandy or ether. Having experimentally given a wineglassful of vinegar several times, without ergot, I have found little benefit to result. Arguing by analogy, I made the following mixture :

℞. Liq. ergotæ ................................
Acid. acetic. concent.......................aa ℥j,
Aether. sulph. (s. g. .735)....................ʒiv.

This should be put into a three-ounce bottle, well corked, and shaken thoroughly. I administer, of this mixture, three teaspoonfuls in a wineglassful of water, and, having used it for a considerable time, I am delighted with its efficacy in causing contraction and giving a refreshing sleep after a short interval, with little or no complaint of after-pains. I combine this with the good old-fashioned pincushion pad wrapped round with a napkin. I

look upon the ordinary binder, with or without the two or three napkins usually offered by the nurse when a pad is asked for, as a delusion and a snare. With the lower edge well below the hips (where it ought to be to keep it from slipping up), the direct pressure on the uterus is almost *nil*, and it serves merely to hinder the pressure of the hand in manipulating the uterus—*Medical Register*.

**The Pupil as a Guide in the Administration of Chloroform.**—1. The effect produced by chloroform on the pupil is at first dilatation, varying in degree and duration, then contraction as the narcosis becomes profound, and dilatation again when the sensibility is returning. If the administration be still continued, with the pupil strongly contracted and motionless, the pupil will also dilate, but in this case more suddenly and completely, and will be coincident with a state from which it will be defficult or impossible to resuscitate the patient. This latter is the dilatation of asphyxia.

2 So long as the pupil dilates in response to excitation by pinching, etc., the patient is not sufficiently narcotized for the operation to be proceeded with, unless the latter is slight and does not require complete anesthesia.

3. When the pupil becomes strongly contracted and immobile, no more chloroform should be given until it begins to dilate again. If, then, further anesthesia be required, a little more chloroform should be given till pupil again contracts.

4. The occurence of sickness causes dilatation similar to, but more sudden than that which happens when sensibility is returning, and the efforts of vomiting have the effect of arousing patients.

The watching of the respiration and the pulse, which are doubtless the best indications of the effect produced on the individual by chloroform, and therefore of vital importance for safe administration, does not in many cases furnish evidence of the state of sensibility, in regard to which he holds the observation of the pupil to be of greatest assistance. The sign most usually relied on, namely, the insensibility of the conjunctiva, is by no means a satisfactory test, for in many cases conjunctival anesthesia is established long before the patient can be said to be under the influence of the drug. By observing the pupil, the administrator of chloroform can tell at once when the effect of the drug is on the wane, because the pupil then begins to dilate slowly. Noticing this, he can, by the administration of a few drops more chloroform till the pupil contracts again, prevent the occurrence of struggling and interruption of rhe operation. In this way he can keep the patient in the state most suitable for the satisfactory performance of the operation without narcotizing him more than is necessary.—*Dr. Nelson in British Medical Journal.*

**Saccharin.**—From half a grain to a grain and a half of saccharin will sweeten a cup of tea or coffee. Small saccharin tablets, indeed, have been prepared for this purpose, and these are now being supplied, it is said, to the German army; for in a small bottle of scarcely appreciable weight a soldier can carry enough saccharin for a week's supply.

There is a wide field for the application of saccharin to the production of sweetmeats and preserves, and in disguising the taste of bitter and nauseous preparations, as well as in preserving the properties of others. But it must always be carefully borne in mind that saccharin is to be regarded as a condiment, and not as a food like sugar, and can, therefore, replace sugar only in exceptional cases.

**Quinine in Gonorrhae.**—Dr. Ledetsch has for three years used injections of quinine in gonorrhea, with brilliant results. The bisulphate one per cent., with glycerine twenty-five, and water seventy-five, was employed, at first three times daily, then twice, and later only once. A slight burning sensation was produced. By this treatment several chronic cases were cured in a few days.

[The editor has combined quinine with the hot water treatment, thirty grains to the half gallon, with good results. We are not tied to the use of ast ingents for urethral injections; indeed, antiseptic injections are displacing them.—A. W. B.]—*Indiana Medical Journal.*

**The Bichloride of Mercury in the Treatment of External Diseases of the Eye.**—Dr. Alt in *American Journal Ophthal.*: —The probability that all diseases of the conjunctiva and lids are due to the action of microbes is very great. Numerous pathogenic (and, as far as known, nonpathogenic) microbes may be found even in the healthy conjunctival sac. In common with others I have, therefore, for a prolonged period tried to find out which of the many germicides now in use and generally approved would be the most suitable in the treatment of the diseases of the lids and conjuctiva. I prefer the bichloride of mercury. The strength in which I have been in the habit of using for over a year varies from one part in 2500 to one part in 5000 (or even in 10,000 in children). Sometimes I found that in cases in which I wanted to use the first named solution, I had to dilute it on account of the severe pain it caused. It is a strange fact that, while most people declare the instillation of a 1 in 2500 solution produces no more disagreeable feeling than a drop of water would cause, others (luckily but few) complain of an excruciating pain following directly upon the instillation. In these latter cases even a weaker solution is usually rather painful.

In *simple conjunctivitis*, with hardly any discharge, but heat and dryness of the lids, especially in the evenings, where boracic acid seems to be useless, instillations of the bichloride of mercury solution morning and evening seem to act well without exception.

In *chronic catarrhal* conjunctivitis its action is very agreeable to most patients, and often no other remedy need be applied.

In all *purulent* forms of conjunctivitis, including the gonorrhœal ones of the new born and adult, the frequent irrigation of the conjunctival sac with a 1 in 2500 solution of the bichloride of mercury seems, in my experience, to be without a rival. Although in some very severe cases I have thought best to apply the nitrate of silver for some days aside from the sublimate solution. The latter I had freely poured into the eyes every half hour, having besides iced compresses applied day and night.

*Ulcers* of the cornea seem to do better when freely irrigated with the bichloride of mercury solution, especially after cauterization with pure carbolic acid. Iodoform, so highly recommended by others, has never given me any satisfactory results.

**Novel Treatment of Hemorrhoids.**—The *Sacramento Medical Times* for January tells the following: "An amusing incident occurred a short time since in one of the northern counties of the State, whereby a granger accidentally discovered an efficient remedy for protruding piles. It seems that 'hog killing' time was at hand, and the granger was busy cutting up and putting away his meat into salt. He had just prepared the mixture with a plentiful supply of black and cayenne pepper, which he had thoroughly mixed together with his hands. Suffering badly from protruding piles, he went into the house and adjusted the offending members without washing his hands. Scarcely had he done so before the cayenne pepper took vigorous hold of the delicate parts, producing reflex contortions, which can be better imagined than described. He says, however, that the piles have not bothered him since, and he is prepared to bet that it will prove an efficacious remedy for the worst cases, if the patient has only the temerity to try it."

**Typhoid Fever and Coffee.**—Dr. H. S. Herr, Westfield, Ind., writes to the *Journal* as follows:

During the last six months there have been a number of typhoid fever cases in this neighborhood; and in reviewing the cases in my own practice and of my neighboring physicians, I notice that nine-tenths of the persons attacked by the fever were those who rarely or never drank coffee. I would like to hear from the readers of your *Journal* on this subject. I have looked upon this as a coincidence, as I do not think that coffee has any preventive qualities as against typhoid. But the fact that persons who drank coffee do not drink as much water as others, and the water in the coffee being boiled, all bacterial life would be destroyed, may account for the coincidence.

**A New Remedy for Night-sweats.**—Dr. Sampson Pope, in a communication to the *Therapeutic Gazette* calls the attention of the profession to an indigenous drug for the relief of night-sweats. He says: "The remedy is one indigeneous to the whole country; it is therefore within the reach of us all; it is the cinquefoil, *potentilla canadensis*, called by some botanists *potentilla sarmentosa.* I have stopped night-sweats with it when atropine failed to relieve. It is pleasant to take; when drawn it has an agreeable odor much like table tea. The manner of preparation is to pour boiling water on a handful of the vine, leaves and root. Let the patient drink *ad libitum*."

"I hope that the members of the profession will give it a fair trial, and report the results through the columns of your valuable journal."

**Diphtheria.**—Dr. J. J. Ashley writes in *Medical Brief* of remedies for diphtheria: I have one that I have used three years, and in many cases without the loss of one. The first case I used it in was a little girl. I found her bolstered up, her mouth open struggling for breath, throat swollen and closed, and the "buckskin" covering the whole throat, the nose having a full share The first teaspoonful of medicine, by the law of gravitation, ran down sides of mouth and I think some got down the throat; at all events the child is here to-day. I have had them where it came out so as to cover the lower lip, extending into the head and nose so that the nostrils were connected with the exudation. And they are here to-day, and besides that likes the doctor for his success. Try the following and report the results through the *Brief*:

NO. 1.

℞. Tinct. Aconiti Rad.............................ʒ ss,

Chloral Hyd..............................ʒ ss,

Glycerini q. s. ad.............................℥ ij.

M. Sig.: Dose, teaspoonful.

NO. 2.

℞. Ammon. Chlor.............................ʒ ij,

Tinct. Belladonnæ.............................ʒ j.

Glycerini q. s. ad.............................℥ ij.

M. Sig.: Teaspoonful.

I give a dose every hour when awake, first No. 1, then No. 2. Give them all the milk they will drink. When the nose is involved I syringe with dil. bromochloralum.

There is one thing about this; you don't frighten your patients, and they take it without compulsion.

**Intestinal Obst uction**—Dr. J. Price exhibited a piece of large intestine which had been removed that day by Dr. Charles B. Penrose. The patient, a woman, had not had a movement of the bowels for twenty-eight days, and there was enormus distension. The constriction was easily found, on the right side of the uterus and posteriorly. The intestine was punctured, and a gallon or more of fæces removed. The two ends of the gut were then united in half their circumference, and the other half was stiched to the abdominal opening, making an artificial anus. Dr. Agnew had seen the patient, and had recommended immediate operation. The woman was a patient of Dr Bernardy's. She was now doing well. The speaker believed that Mr. Tait had first advised the preliminary puncture to relieve the distension in conditions that could not be delt with by resection. He had some letters from Dr. McMurtry, of Danville, Ky., who had been called to see a physician, twenty-six years old. On opening the abdomen, he found two perforating ulcers of the cæcum with local peritonitis He had trimed the edges of the ulcers, and closed them with Lembert sutures ; irrigation and drainage was used. On the fourth day the pulse was 92, the temperature was 99° F., and the patient had a complete evacuation of the bowels.—*New York Medical Journal.*

**Tincture Iodine in Persistent Vomiting During Labor.**—Dr. W. T. Dodge, in the *Medical Record* for Dec. 24, advises for persistent vomiting after labor tincture iodine in five drop doses. He reports a case, and after having used lactopeptine, dilute hydrocyanic acid, carbolic acid, sub. nit bismuth, lime water, etc., etc., without relief, the administration of five drop doses of tincture iodine gave immediate and permanent relief. He gives Dr. Eliot credit for the suggestion, who, in an article in the *Quart. Comp. of Medical Sciences* for October, gives a very good resume of the effect of this remedy, and gives quite a number of authorities either for or against the use of the tincture iodine internally, and reports one case where persistent vomiting during a tedious labor was checked as by magic by five drops of tincture iodine in a tablespoonful of sweetened water. He repeated the dose in thirty minutes to prevent a return of the vomiting—*Texas Courier-Record.*

**Tar.**—Some months ago the floors of many Austrian garrisons were painted with tar, and the results have proved so uniformly advantageous that the method is becoming greatly extended in its application. The collection of dust in cracks is thus prevented, and a consequent diminution in irritative diseases of the eye has been noted. Cleanliness of the rooms has been greatly facilitated, and parasites are almost completely excluded. The coating of tar is inexpensive, requires renewal but once a year, and presents but one disadvantage, namely, its somber color.

**Cocaine in Gonorrhea.**—Of all the twisting, burning, lancinating pains which afflict mankind, that accompanying the act of micturition during the acute stage of a gonorrhea is perhaps the worst. A great point is gained if, in some simple way, this excess of pain can be obviated, and an injection of four per cent. solution of cocaine overcomes the difficulty almost completely. Before micturition, let the patient inject from half a dram to a dram, retain it for a few minutes, after which micturitition can be accomplished painlessly.—*Medical Review.*

**Effects of Tea.**—Dr. Bullard, of Boston, has lately published some studies on the effects of tea. Among other things he points out the fact that the action of tea is cumulative, and its effects are first seen in the weak and ailing. The amount necessary to produce toxic symptoms is rather under five cups of average strength daily. The symptoms of chronic tea poisoning are dyspepsia, palpitations, nervous and neuralgic affections. If the habit were attended to with onset of the dyspeptic symptoms graver results might be averted.

